# Prurigo Pigmentosa: A Clinicopathological Report of Three Middle Eastern Patients

**DOI:** 10.1155/2018/9406797

**Published:** 2018-07-09

**Authors:** N. Almaani, A. H. Al-Tarawneh, H. Msallam

**Affiliations:** ^1^Department of Dermatology, Faculty of Medicine, The University of Jordan, Amman, Jordan; ^2^Department of Dermatology, Jordan University Hospital, Amman, Jordan; ^3^Department of Dermatology, Faculty of Medicine, Mu'tah University, Karak, Jordan

## Abstract

Prurigo pigmentosa is a unique cutaneous inflammatory disorder characterized by a sudden onset of pruritic and erythematous macules, urticarial papules, and plaques that may coalesce to form a reticulated pattern. Lesions typically heal within weeks leaving a reticulated and mottled postinflammatory hyperpigmentation. The majority of reported cases originate from Japan with much fewer cases described worldwide without predominant ethnicity. The histopathological features of prurigo pigmentosa can be nonspecific; however, distinct features exist for each stage of the disease. The aetiology of prurigo pigmentosa is not fully understood. However, ketoacidosis has been implicated in the pathogenesis and indeed prurigo pigmentosa has been associated with ketoacidotic states such as diabetes mellitus, fasting, dieting, and anorexia nervosa. In this report, we present 3 Jordanian patients with prurigo pigmentosa and describe their clinicopathological features. One patient developed prurigo pigmentosa while fasting during the month of Ramadan and another was undertaking a strict diet. No associations were identified in the third patient. In view of the largely nonspecific clinical and histological features, a high index of suspicion is required as many cases of prurigo pigmentosa are probably undiagnosed.

## 1. Introduction

Prurigo pigmentosa (PP) is a unique cutaneous inflammatory disorder first described in Japan by Nagashima* et al.* in 1971 as a “peculiar pruriginous dermatosis with gross reticular pigmentation.” [1] The term “prurigo pigmentosa” was later coined in 1978. [2] PP is an under-recognized disorder in countries other than Japan, where hundreds of cases have been reported. On the contrary, much fewer cases have been described worldwide without predominant ethnicity [3–6]. Herein, we present 3 Jordanian patients with PP and describe their clinicopathological features.

## 2. Case Presentation

**Figure 1:**
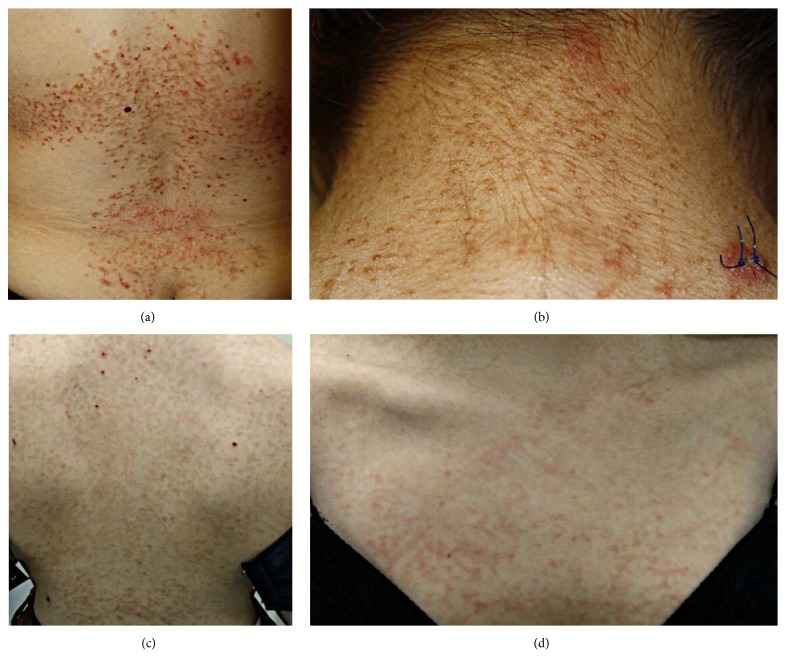
**Clinical features of prurigo pigmentosa.** Patient 1 was noted to have symmetrically distributed erythematous papules and papulovesicules, admixed with postinflammatory and reticulated hyperpigmentation on the middle back, lumbosacral area, lateral chest wall (a), and the nape of the neck (b). In patient 2, there were erythematous papules with postinflammatory hyperpigmentation on the lateral chest wall, the back (c), and the central chest (d). Scattered excoriations were also noted.

Patient 1 is a 31-year-old Jordanian female with a history of a recurrent and itchy eruption involving the mid- to lower back, lateral chest wall, and the nape of the neck. This resolved with net-like pigmentation (Figures 1(a) and 1(b)). The occurrence of the eruption was linked with fasting in Ramadan, in addition to travels to North America. No other medical problems were identified.

Patient 2 is a 16-year-old Jordanian female who presented with an itchy eruption of new onset. This appeared 3 weeks earlier and affected the upper to mid-back and the “V” of the neck (Figures 1(c) and 1(d)). The occurrence of the eruption followed a 1-month period of strict dieting.

Patient 3 is a 45-year-old Jordanian female with an itchy eruption of 3 months' duration. This affected the nape of the neck and the upper back. No triggers were identified and the patient was otherwise healthy.

The patients' demographics and their clinical features are outlined in Table 1. Clinically, all patients were noted to have erythematous papules that coalesced to form plaques. These were arranged in a reticular pattern that was more prominent peripherally. In addition, patient 1 had associated vesicles and minimal erosions (Figures 1(a) and 1(b)). In all patients, the lesions were symmetrically distributed and had a predilection for the trunk. Other involved areas included the lateral and posterior aspects of the neck (patients 1 and 3), the lateral chest wall (patient 1), and the lumbosacral area (patient 1). Different types of lesions coexisted in all patients including papules, patches, and plaques, in addition to vesicles and erosions in patient 1. A clinical diagnosis of PP was suspected clinically in patients 1 and 2.

**Figure 2:**
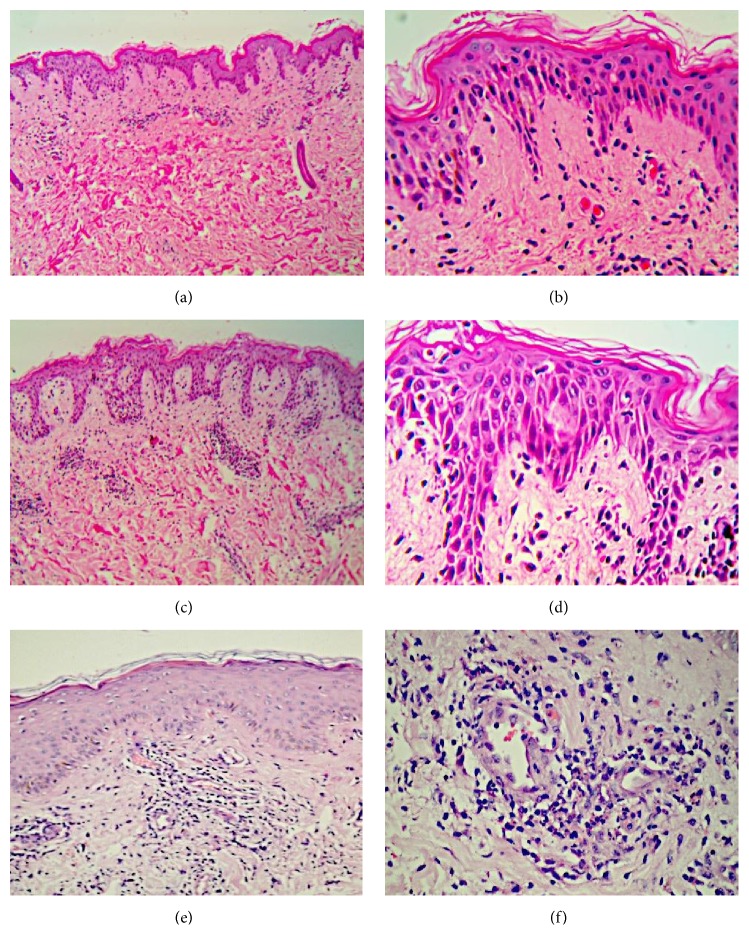
**Histopathological features of prurigo pigmentosa.** Biopsies taken showed evidence of mild hyperkeratosis, acanthosis, spongiosis, and mild superficial perivascular inflammatory cell infiltrate on low power in patient 1, patient 2, and patient 3 (a, c, and e, resp.), as well as dyskeratosis and hydropic degeneration of the basal cell layer, papillary dermal oedema, and superficial perivascular lymphoid cell infiltrate containing neutrophils and nuclear dust on high power. High power showed additional features of dyskeratotic hydropic degeneration of the basal cell layer, papillary dermal oedema, and superficial perivascular lymphoid cell infiltrate containing neutrophils and nuclear dust in patient 1, patient 2, and patient 3 (b, d, and f, resp.).

The main histological findings are summarized in Figure 2 and Table 2. The histopathological features were similar in all cases, showing features consistent with early lesions according to Boer's criteria [7]. The major histological differential diagnoses were impetiginized spongiotic dermatitis, pityriasis lichenoides, and viral exanthem. Periodic acid-Schiff stain was negative in all specimens. Direct immunofluorescence was performed for patients 1 and 2 only and was negative.

The clinical course varied, but all three patients had eventual complete resolution of all lesions. Patient 1 was treated with superpotent topical corticosteroids prior to presentation to our department. However, there was no improvement and new lesions continued to emerge. The patient subsequently reported gradual spontaneous resolution 10 weeks after onset of the eruption, leaving postinflammatory hyperpigmentation. Patient 2 was previously treated with moderately potent topical corticosteroids and antihistamines without any improvement. New lesions continued to emerge. On initiation of doxycycline, the lesions cleared within 1 week. No recurrence was reported during a 10-month follow-up period throughout which the patient avoided strict dieting. Patient 3 reported spontaneous resolution of some lesions before presentation to our department. Doxycycline was subsequently initiated with complete resolution.

## 3. Discussion

Prurigo pigmentosa continues to be described more frequently in Japanese patients, yet reports have emerged from other countries, albeit in much smaller numbers [3, 4, 6]. This might reflect underreporting or misdiagnosis rather than a genetic predilection for the Japanese population [3, 5, 8]. PP most commonly occurs in females in the third decade of life (range: 7-61 years) with a female-to-male ratio of 2-4:1 [3, 4, 9]. This is consistent with the findings in our report, where all patients were female with a mean age of 31 years.

Seasonal clustering is reported in the literature particularly in the spring and summer [3, 4], as in our cases. Reported cases were sporadic with no reported familial clustering [8].

PP is characterized by a sudden onset of pruritic and erythematous macules, urticarial papules, and plaques that may coalesce to form a reticulated pattern [4, 8–10]. Pustular and bullous variants have been reported [3, 4, 10]. Scales and crusts usually appear while the lesions are resolving [3, 4]. Complete resolution might take from one to several weeks [4, 10]. However, lesions typically heal with reticulated and mottled postinflammatory hyperpigmentation that usually persists for months [3, 4, 9, 10].

PP typically has a symmetrical distribution with a predilection for the nape of the neck, central chest, upper back, lumbosacral area, and abdomen [3–5, 11]. However, asymmetric patterns have been described including unilateral [12] and segmental [11] distributions. On the chest, the inter- and submammary areas are most frequently affected [3]. Involvement of the hair, nails, and mucous membranes has not been described [3, 8]. Recurrences are common in the course of this disease and might occur months or years after initial presentation [8]. The three patients described in this report exhibited clinical features consistent with those described in the literature, with lesions of various stages of development noted at the time of presentation.

The aetiology of PP is not fully understood. However, endogenous and exogenous factors have been implicated including atopic diathesis, Sjogren's disease, and adult onset still's disease [3–5, 13].

A possible hormonal role has been hypothesized as worsening during pregnancy and menstruation has been reported [3, 5]. Multiple infectious agents such as* Helicobacter pylori* and* Borrelia spirochetes* may have associations with PP [3]. Possible aggravating exogenous factors include sweat, summer heat [4, 5], sun light [3, 5], physical trauma, friction [9, 12, 14], and contact allergens [3, 13]. Moreover, due to the recurrent nature of PP, a viral association has been postulated; however, this has not been confirmed [9].

More recently, the role of ketoacidosis in the pathogenesis of PP has gained momentum. This occurs with diabetes mellitus, fasting, dieting, anorexia nervosa, and following bariatric surgery, all of which are associated with PP [3–5, 9, 14]. Many studies documented a high level of ketones in the blood or urine [3, 7, 14]. Ketone bodies are thought to accumulate around blood vessels, leading to a predominantly neutrophilic inflammation [7]. The ketones subsequently enter the cells, leading to alterations in intracytoplasmic cellular processes [7]. In our report, patient 1 developed PP while fasting during Ramadan, while patient 2 was undertaking a strict diet. Unfortunately, ketone levels were not measured. In recent papers, PP cases associated with ketogenic diet were successfully treated with diet correction only [15]. In those cases, the efficacy of antibiotic therapy is probably due to the improvement on gut microbiome.

The histopathological features of PP can be nonspecific; however, distinct features exist for each stage of the disease [3–5, 7, 8]. The early stage is characterized by a superficial and perivascular dermal neutrophilic infiltrate along with papillary dermal edema, slight spongiosis, and neutrophilic exocytosis. The following stage, where lesions are fully developed, is characterized by a heavier dermal infiltrate in a lichenoid pattern. Lymphocytes usually predominate over neutrophils and the epidermis shows a variable degree of spongiosis, reticular degeneration of basal cell layer, and numerous necrotic keratinocytes. In the final resolution stage, a sparse lymphocytic dermal infiltrate is found along with upper dermal melanophages. The epidermis becomes hyperplastic with focal parakeratosis and few scattered necrotic keratinocytes. All our three cases showed similar histopathological features as seen in Figure 2. Boer* et al.* proposed that the histopathological changes of prurigo pigmentosa may be specific and transpire rapidly [16]. The histopathological features of our cases concur with this proposal. Therefore, the histopathological features of prurigo pigmentosa are diagnostic in the appropriate clinical setting.

Histological differential diagnoses include spongiotic dermatitis with secondary impetiginization, early guttate psoriasis, viral exanthem, and acute spongiotic dermatitis in the fully developed stage and postinflammatory hyperpigmentation and chronic spongiotic dermatitis in the late stage [3]. In addition, it has been suggested that both PP and confluent and reticulated papillomatosis of Geougerot and Carteaud lie on a spectrum of one disease [13]. Direct immunofluorescence studies have consistently been reported as either negative or nonspecific [3, 8, 11]. Direct immunofluorescence studies were only done for patients 1 and 2 and were negative.

In view of the largely nonspecific clinical features, the diagnosis of PP requires a high index of suspicion, as well as clinicopathological correlation. Clinical differential diagnoses include acute lupus erythematosus, dermatitis herpetiformis, linear immunoglobulin A disease, pigmented contact dermatitis, confluent and reticulated papillomatosis of Geougerot and Carteaud, Dowling-Degos disease, macular amyloidosis, and ashy dermatosis [3–5, 8].

Multiple therapeutic options exist for PP, yet tetracyclines remain the favoured option. This is thought to be related to their anti-inflammatory effect, particularly in the inhibition of neutrophil migration and function, matrix metalloprotease activity, and proinflammatory cytokine expression [3–5, 8, 10].

Other therapeutic options include macrolide antibiotics, dapsone, sulfamethoxazole, isotretinoin, and potassium iodide [3–5, 8–10, 14]. Corticosteroids and antihistamines have limited, if any, effect on PP [3, 5, 9], helping to differentiate PP from steroid-responsive dermatoses such as eczema.

The two patients who received doxycycline in this report had rapid clearance of the eruption. Patients were followed up for at least 6 months with maintained clearance. However, patients were advised about possible recurrence with future fasting or dieting, as well as other ketotic states.

To our knowledge, this is the first report of PP in Jordanian patients. The paucity of reports outside Japan is likely attributed to lack of awareness and misdiagnosis. Clinicopathological correlation is imperative in making this diagnosis as is the awareness of the possible triggering factors including ketoacidotic states such as fasting and strict diets.

## Figures and Tables

**Table 1:** Demographics, clinical features, and outcomes of 3 Jordanian patients with prurigo pigmentosa.

	**Patient 1**	**Patient 2**	**Patient 3**
**Age (years)**	32	16	45
**Gender**	Female	Female	Female
**Symptoms**	Recurrent itchy eruption	Itchy eruption	Itchy eruption
**Distribution**	Lateral and nape of the neck, mid and lower back, lateral chest wall	Upper and mid back, V-area of the chest	Nape of the neck and upper back
**Duration of lesions**	1 year, recurrent	3 weeks	1 month
**Clinical examination**	Reticulated erythematous papulovesicular lesions with focal erosions and crusting, prominent postinflammatory hyperpigmentation	Erythematous maculopapular lesions with faint postinflammatory hyperpigmentation	Erythematous urticarial reticulated papular lesions
**Triggers**	Ramadan fasting	Strict dieting	No reported association
**Treatment**	Spontaneous resolution	Doxycycline 100mg po bid for 1 month	Doxycycline 100mg po bid for 1 month
**Follow-up**	No recurrences during 10 month follow up period	Excellent response within 2 weeks, no recurrences after stopping strict diets	Excellent response, no recurrences after treatment

**Table 2:** The main histologic findings in 3 Jordanian patients with prurigo pigmentosa.

**Lesion biopsied**	**Patient 1**	**Patient 2**	**Patient 3**
	Papulovesicular neck lesion	Erythematous papule on the back	Erythematous urticated plaque on the neck
**Epidermal changes**			
Orthokeratosis	+	+	+
Parakeratosis	−	−	−
Scale crust	−	+	−
Epidermal hyperplasia	+	+	+
Keratinocyte necrosis	+	+	+
Spongiosis	+	+	+
Vesiculation	−	−	−
Neutrophilic exocytosis	−	+	−
Basal cell vacuolization	+	+	+
Secondary impetiginization	−	−	−

**Dermal changes**			
Perivascular lymphocyte infiltrate	+	+	+
Perivascular polymorphonuclear infiltrate	+	+	+
Papillary dermal oedema	+	+	+
Pigment incontinence	−	−	−
